# Pulmonary manifestations of long COVID in Johannesburg, South Africa

**DOI:** 10.4102/sajid.v40i1.734

**Published:** 2025-10-17

**Authors:** Charles Feldman, Ncomeka Manentsa, Godspower Akpomiemie, Andile Jabavu, Karlien Moller, Esther Baskhar, Bukelwa Mtshazo, Ememabasi Edem, Simiso M. Sokhela, Samanta T. Lalla-Edward, Willem D.F. Venter, Guy A. Richards

**Affiliations:** 1 Department of Internal Medicine, Faculty of Health Sciences, University of the Witwatersrand, Johannesburg, South Africa; 2 Wits Ezintsha, Faculty of Health Sciences, University of the Witwatersrand, Johannesburg, South Africa; 3 Department of Surgery, Faculty of Health Sciences, University of the Witwatersrand, Johannesburg, South Africa

**Keywords:** 6-minute walk test, COVID-19 infection, high-resolution tomographic scan, long COVID, spirometry, symptoms

## Abstract

**Background:**

There are few studies of long coronavirus disease (COVID) in low- and middle-income countries.

**Objectives:**

This study investigated long-term pulmonary manifestations of long COVID among adults in Johannesburg, South Africa.

**Method:**

This was a respiratory sub-study of a larger long COVID investigation. Cases with self-reported long COVID symptoms were recruited into four cohorts: prior asymptomatic infection, mild to moderate infection, hospitalised for severe infection and vaccinated prior to infection. Cases with respiratory comorbidity and/or well-characterised exposure to certain conditions (e.g. cigarette smoking) were excluded. Demographics, clinical features, spirometry, six-minute walk test (6MWT) and high-resolution computerised tomographic (HRCT) scan of the chest were recorded.

**Results:**

Of the 171 patients interviewed from the initial study, 36 with appropriate inclusion criteria were recruited a median of 2.1 years following their acute COVID-19 illness. Accordingly, the incidence of long COVID was 21.1% (36/171 patients) for the group as a whole and 5.9% (3/51), 25.0% (14/56), 37.8% (17/45) and 10.5% (2/19) for cohorts 1–4, respectively (*p* = 0.001). The major symptoms were tiredness and/or fatigue, shortness of breath and cough. Overall, 33 patients had abnormal 6MWT results, and 10 had abnormalities on spirometry; obstructive pattern in five, restrictive in three and mixed in two. Seven patients (six of whom were previously hospitalised) had probable/possible abnormalities compatible with long COVID on HRCT scan (*p* = 0.045).

**Conclusion:**

This study documented respiratory abnormalities in patients as long as 2 years after prior SARS-CoV-2 infection, especially among those with severe prior infection.

**Contribution:**

This was among the first studies comprehensively documenting pulmonary abnormalities in patients with long COVID in South Africa.

## Introduction

Post-coronavirus disease 2019 (COVID-19) condition (long COVID) is a syndrome characterised by persistent symptoms and/or delayed or long-term complications following infections with the severe acute respiratory syndrome coronavirus 2 (SARS-CoV-2); however, there have been many definitions and various attempts at consensus since its recognition.^1,2,3^

One of the most common organ systems involved in long COVID is the lung, with publications describing pulmonary symptoms, lung parenchymal changes and functional abnormalities.^1,2,3,4,5,6,7,8,9,10^ The common respiratory symptoms are fatigue, dyspnoea, chest pain/tightness and cough, which in one systematic review and meta-analysis were documented in 52%, 37%, 16% and 14% of patients, respectively, from 3 weeks to 3 months following hospital discharge following COVID-19 infection.^8^ Parenchymal abnormalities have been noted in the lungs of patients with long COVID using high-resolution computerised tomographic (HRCT) scan of the chest.^5,6^ These changes manifest variously as restrictive, obstructive or combined restrictive and obstructive patterns on spirometry, altered diffusion capacity of the lungs for carbon monoxide (DLCO) and reduced six-minute walking distance (6MWD).^9^ In addition, decreased functional capacity and health-related quality of life have been described, in one study among 36% and 52% of cases, respectively.^10^

Upon review of the different studies of the pulmonary manifestations of long COVID, it is apparent that methodologies used were not uniform.^4,5,6,7,8,9,10^ Few mentioned pulmonary comorbidities (e.g. asthma or chronic obstructive pulmonary disease [COPD]) or risk factors for lung abnormalities existing prior to the acute COVID-19 illness (e.g., smoking, biomass fuel exposure or past tuberculosis), which may have accounted for the abnormalities documented. Furthermore, even if pre-existing conditions were found in previous studies, few of these studies controlled for them, to exclude potential confounding. Timing of the various investigations following acute COVID-19 infection has also varied from just over 4 weeks to 2 years or more.^11,12,13,14,15,16,17^ At the time of this study, we were unaware of any comprehensive investigations of pulmonary manifestations of long COVID in sub-Saharan Africa, especially in South Africa.

The aim of this sub-study was to investigate the long-term pulmonary manifestations of long COVID in a South African population that had recovered from acute COVID-19 infection of various degrees of severity.

## Research methods and design

This was a sub-study of a larger observational study of long COVID undertaken at Ezintsha Research Centre in Johannesburg, between May 2020 and July 2022. Patients had been allocated to one of four cohorts: (1) those with previous asymptomatic COVID-19 infection (cohort 1) were patients who were tested for COVID-19 infection for various reasons, in the absence of any symptoms, and tested positive, (2) those with previous mild to moderate infection (cohort 2) were those with the presence of symptoms, which may have led to a medical consult but did not require management in hospital, (3) those with previous severe infection (cohort 3) were cases with more severe symptoms, requiring patient management in hospital and (4) these were patients who were vaccinated in a clinical trial conducted in 2020, prior to the occurrence of their COVID-19 infection (cohort 4).

This respiratory sub-study enrolled cases between June 2023 and January 2024. The definition used for long COVID was the continuation, or development of new symptoms, at least 3 months after the initial SARS-CoV-2 infection, with these symptoms lasting for at least 2 months and with no other explanation, according to the recommendations of the World Health Organization (WHO),^18^ which was the commonly used definition at the time this sub-study began.

Participants were adults (≥18 years) contacted from the main study, who were willing to participate in the sub-study. They needed to be able to sign informed consent and perform lung function testing and were recruited approximately 2 years after their acute infection. Current or previous smokers, those with a well-characterised history of household exposure to biomass fuel, workplace chemicals and/or dust, previous diagnosis of tuberculosis or a well-documented comorbid respiratory illness, such as asthma, COPD or other chronic respiratory condition, were excluded. Procedures performed on each patient are showed in Online Appendix Table 1-A1. Women of child-bearing age were tested for pregnancy and if pregnant were excluded.

Study procedures included a detailed evaluation of long COVID symptoms. Those symptoms spontaneously reported by the patients were considered the primary symptoms, and those elicited on further questioning were reported as secondary symptoms. Evaluation of dyspnoea was undertaken using the modified Medical Research Council Dyspnoea Scale (mMRC).^19^ A clinical examination was conducted, including measurement of the body mass index (BMI). Lung function studies included spirometry (pre- and post-bronchodilator), a six-minute walk test (6MWT) and an HRCT scan of the chest. The latter was performed at a private Medical Centre, using standard HRCT protocols and reported by a qualified radiologist. Lung function studies and HRCT scans were reviewed by two experienced pulmonologists on the study team (C.F. and G.A.R.). Measurements of thoracic gas volumes and diffusion capacity were planned but were not able to be performed.

Lung function testing was performed using equipment approved by, and performed according to, the American Thoracic Society/European Respiratory Society criteria.^20,21,22,23,24,25^ The algorithm used for measurement of the normal 6MWT values was that described by Jenkins et al., which appeared to be the most suitable for the study population.^25^ Oxygen saturation was measured using a portable oximeter.

Patients with findings requiring further evaluation and/or management by a pulmonologist were referred to the Charlotte Maxeke Johannesburg Academic Hospital Respiratory Clinic, as previously arranged.

### Statistical analysis

Data were captured directly onto REDCap for electronic data source and transferred to Stata 18 software for data management and analysis. The analysis was descriptive and focused on differences in demographic, clinical and laboratory characteristics, across all four cohorts. The Kruskal–Wallis test was used to compare the medians of continuous variables, and chi-squared and Fisher’s exact tests were used for categorical variables. Statistical significance of *p* < 0.05 applied.

### Ethical considerations

Ethical clearance to conduct this study was obtained from the University of the Witwatersrand, Human Research Ethics Committee (Medical) (approval number: 220304B), and all participants signed individual consent to participate in the study. The study was conducted in accordance with the Declaration of Helsinki.

## Results

**FIGURE 1:**
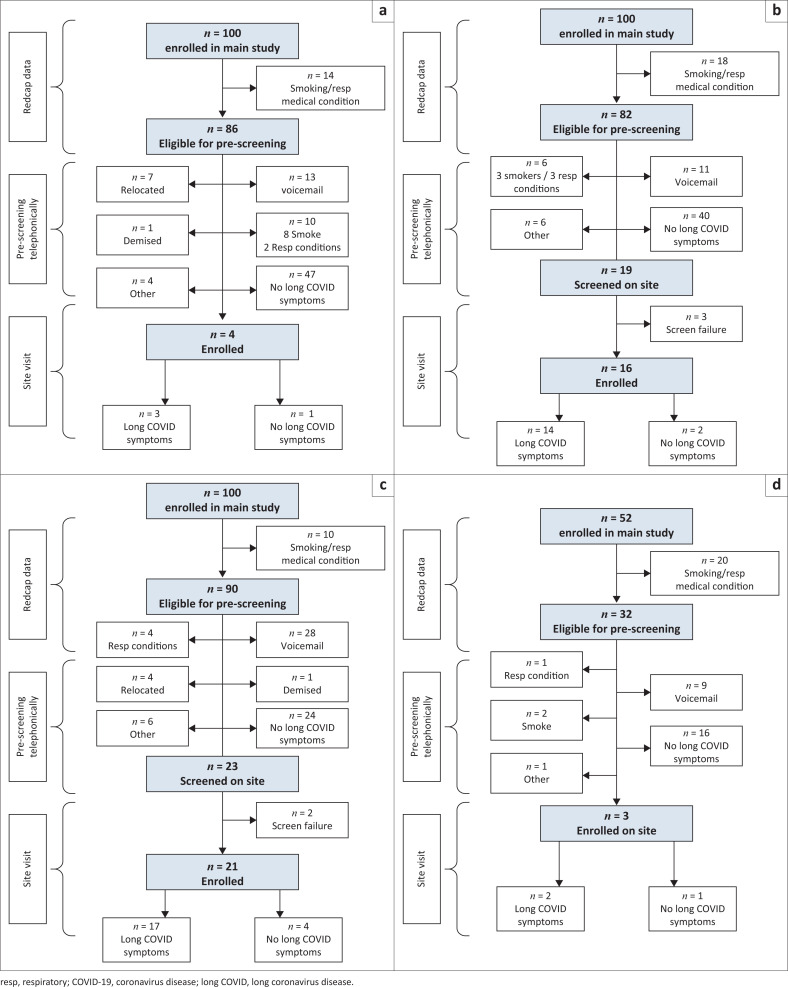
Screening for eligibility for participation in the respiratory sub-study of long coronavirus disease (COVID) in patients following an episode of COVID-19 infection. (a) Cohort 1: Asymptomatic COVID-19 infection. (b) Cohort 2: Mild to moderate symptoms of COVID-19. (c) Cohort 3: Severe symptoms of COVID-19. (d) Cohort 4: Vaccinated prior to COVID-19.

From the parent study, 352 patients were screened for the sub-study. Several were excluded, including smokers and/or patients with underlying respiratory conditions or risk factors (85), unreachable patients (74) and others for various reasons (22) (Figure 1). Thus, 171 patients were evaluable for long COVID of whom 36 had self-reported symptoms, a median of 2.1 years (range 1.7 to 2.7 years) following their acute COVID-19 illness. The incidence of long COVID was, therefore, 21.1% (36/171 patients).

### General characteristics of the study cohorts

Among the cohort that had previously asymptomatic acute infection, 5.9% (3/51 patients) had long COVID symptoms, while 25% (14/56) of those with previous mild-moderate infection, 37.8% (17/45) of those with previous severe infection and 10.5% (2/19) of those with prior vaccination had long COVID symptoms (*p* = 0.001) (Figure 1).

Demographics, clinical characteristics and respiratory parameters among patients in the four cohorts are shown in Table 1. The median age of the patients, of whom four were males (11%) and 32 females (89%), was 47 years (interquartile range [IQR]: 38, 59). The median BMI was 34.0 kg/m^2^ (IQR: 30.0, 41.6) (Table 1). Common comorbidities were hypertension and diabetes mellitus with a trend for the former to be more common in cohort 3 (*p* = 0.068). Online Appendix Table 2-A1 shows the symptoms that the patients reported. The major primary and secondary symptoms were tiredness/fatigue, shortness of breath and cough. The mMRC score was increased to ≥ 1 in 24 cases, most frequently in cohort 3 (*p* = 0.030) (Table 1).

**TABLE 1:** Demographic and clinical characteristics of 36 patients with long COVID symptoms.

Variable	Total (*N* = 36)				Asymptomatic (*n* = 3)				Non-severe (*n* = 14)				Severe (*n* = 17)				Vaccinated (*n* = 2)				*p*
	Median	IQR	*n*	%	Median	IQR	*n*	%	Median	IQR	*n*	%	Median	IQR	*n*	%	Median	IQR	*n*	%	
Age†	47	38a, 59	-	-	44	31, 47	-	-	45	34, 56	-	-	52	43, 62	-	-	42	38, 46	-	-	0.209
Female‡	-	-	32	88.9	-	-	2	66.7	-	-	14	100.0	-	-	14	82.4	-	-	2	100.0	0.190
BMI (kg/m^2^)†	34.0	30.0, 41.6	-	-	31.5	29.3, 32.5	-	-	33.2	29.9, 41.6	-	-	37.0	31.4, 42.5	-	-	30.0	20.2, 39.9	-	-	0.412
Hypertension‡	-	-	11	30.6	-	-	1	33.3	-	-	2	14.3	-	-	8	47.1	-	-	0	0	0.068
Diabetes mellitus‡	-	-	4	11.1	-	-	0	0	-	-	0	0	-	-	4	23.5	-	-	0	0	0.107
Comorbidity – other‡	-	-	4	11.1	-	-	0	0	-	-	0	0	-	-	4	23.5	-	-	0	0	0.107
mMRC score ≥ 1‡	-	-	24	66.7	-	-	2	66.7	-	-	6	42.9		-	15	88.2	-	-	1	50	0.030*
6MWD†	445.3	370.6, 530.0	-	-	507.0	415.1, 508.5	-	-	423.9	354.2, 539.5	-	-	455.1	410.0, 477.0	-	-	516.0	376.2, 655.8	-	-	0.412
Abnormal HRCT scan of chest‡	-	-	8	22.2	-	-	0	0	-	-	1	7.1	-	-	7	41.2	-	-	0	0	0.045*
Patients with Long COVID‡	-	-	36/171	21.1	-	-	3/51	5.9	-	-	14/56	25.0	-	-	17/45	37.8	-	-	2/19	10.5	0.001*
COVID infection to sub-study (days)†	783	639, 962	-	-	749	649, 828	-	-	699	628, 892	-	-	951	774, 972	-	-	749	718, 780	-	-	0.343

Note: The 36 cases were divided into four cohorts, based on the severity of their prior COVID-19 infection (cohorts 1–3) or whether they had been vaccinated prior to their prior COVID-19 infection (cohort 4).

6MWD, 6-minute walking distance; BMI, body mass index; HRCT, high-resolution computerised tomographic; IQR, interquartile range; mMRC, Modified Medical Research Council Dyspnoea Scale; IQR, interquartile range.

* , *p* < 0.05 denote statistical significance.

† , Continuous variables: Kruskal–Wallis test was used to compare medians across all four cohorts;

‡ , Categorical variables: Chi-squared and Fisher’s exact test were used to compare groups.

### Lung function measurements in the individual patient cohorts

#### Spirometry

The results of the pre- and post-bronchodilator spirometry are shown in Table 2. The quality of all flow-volume loops (FVLs) was adequate, except for one in cohort 2, which was, therefore, excluded from the analysis. In cohort 1, the FVL of one patient was normal, another showed obstructive lung disease (normal forced expiratory volume in 1 second [FEV1] and forced vital capacity [FVC] but with an FEV1/FVC ratio of 0.62 with a 12% [plus 200 mL] reversibility of the FEV1, as well as a reduced forced expiratory flow between 25% and 75% of the expiratory curve [FEF 25% – 75%] with 26% reversibility following administration of a bronchodilator [41% to 52% predicted]) and the third showed restrictive changes together with reversible obstructive lung disease (FEV1 reversibility of 16% [plus 260 mL] and a reduced FEF 25% – 75% with 18% reversibility following bronchodilator [55% to 65% predicted]).

**TABLE 2:** Spirometry measurements in 36 patients with long COVID symptoms.

Variable	Total (*N* = 35)				Asymptomatic (*n* = 3)				Non-severe (*n* = 13)				Severe (*n* = 17)				Vaccinated (*n* = 2)				*p*
	Median	IQR	*n*	%	Median	IQR	*n*	%	Median	IQR	*n*	%	Median	IQR	*n*	%	Median	IQR	*n*	%	
**Pre-bronchodilator**																					
FVC (L)†	3.05	2.65, 3.60	-	-	4.28	2.01, 4.41	-	-	3.10	2.86, 3.60	-	-	2.92	2.64, 3.16	-	-	2.70	2.65, 2.74	-	-	0.438
FVC%†	98.65	88.29, 112.73	-	-	87.50	64.01, 125.15	-	-	103.39	93.33, 116.73	-	-	101.73	89.03, 108.55	-	-	86.59	80.30, 92.88	-	-	0.505
FVC < 80%‡	-	-	6	17.1	-	-	1	33.3	-	-	2	15.4	-	-	3	17.6	-	-	0	0	0.766
FEV1 (L)†	2.50	2.09, 2.77	-	-	2.66	1.64, 3.91	-	-	2.65	2.45, 2.77	-	-	2.39	2.09, 2.54	-	-	2.12	2.06, 2.19	-	-	0.329
FEV1%†	97.21	86.56, 106.54	-	-	90.48	60.74, 92.65	-	-	101.53	91.47, 110.09	-	-	99.20	92.10, 104.37	-	-	79.42	72.28, 86.56	-	-	0.120
FEV1 < 80%‡	-	-	7	20.0	-	-	1	33.3	-	-	2	15.4	-	-	3	17.6	-	-	1	50.0	0.472
FEV1/FVC ratio†	0.82	0.78, 0.88	-	-	0.82	0.62, 0.89	-	-	0.81	0.78, 0.86	-	-	0.83	0.79, 0.88	-	-	0.79	0.78, 0.80	-	-	0.645
FEV1/FVC < 0.70‡	-	-	1	2.9	-	-	1	33.3	-	-	0	0	-	-	0	0	-	-	0	0	0.143
PEFR L/s†	6.47	5.88, 7.74	-	-	6.95	5.30, 12.05	-	-	6.88	6.13, 7.60	-	-	6.47	6.09, 7.74	-	-	5.42	4.96, 5.88	-	-	0.352
PEFR%†	103.26	94.32, 119.44	-	-	101.61	81.66, 124.23	-	-	105.52	96.00, 121.93	-	-	106.41	95.61, 113.41	-	-	83.69	79.23, 88.16	-	-	0.217
PEFR% < 80%‡	-	-	3	8.6	-	-	0	0	-	-	1	7.7	-	-	1	5.9	-	-	1	50.0	0.322
FEF25-75†	86.33	70.18, 98.51	-	-	54.91	40.87, 101.04	-	-	80.28	76.04, 90.54	-	-	91.72	78.67, 105.05	-	-	55.66	48.63, 62.69	-	-	0.146
FEF25-75 < 65%‡	-	-	7	20.0	-	-	2	66.7	-	-	1	7.7	-	-	2	11.8	-	-	2	100.0	0.008*
**Post-bronchodilator**																					
FVC (L)†	3.07	2.65, 3.41	-	-	4.22	2.27, 4.45	-	-	3.12	2.80, 3.25	-	-	3.00	2.65, 3.29	-	-	2.66	2.55, 2.77	-	-	0.511
FVC%†	103.17	88.33, 111.52	-	-	83.73	72.29, 130.12	-	-	102.79	94.55, 112.73	-	-	104.76	92.21, 111.46	-	-	85.59	77.27, 93.90	-	-	0.543
FVC < 80%‡	-	-	6	17.1	-	-	1	33.3	-	-	2	15.4	-	-	2	11.8	-	-	1	50.0	0.316
FEV1 (L)†	2.50	2.13, 2.83	-	-	2.98	1.90, 3.71	-	-	2.71	2.45, 2.79	-	-	2.45	2.04, 2.71	-	-	2.21	2.13, 2.29	-	-	0.445
FEV1%†	101.36	88.97, 106.84	-	-	87.91	70.37, 101.36	-	-	103.83	94.58, 111.42	-	-	102.45	94.07, 106.11	-	-	82.63	74.74, 90.51	-	-	0.151
FEV1 < 80%‡	-	-	7	20.0	-	-	1	33.3	-	-	2	15.4	-	-	3	17.6	-	-	1	50.0	0.472
FEV1/FVC ratio†	0.84	0.81, 0.87	-	-	0.84	0.67, 0.88	-	-	0.85	0.81, 0.89	-	-	0.83	0.82, 0.86	-	-	0.83	0.83, 0.84	-	-	0.972
FEV1/FVC < 0.70‡	-	-	2	5.7	-	-	1	33.3	-	-	0	0	-	-	1	5.9	-	-	0	0	0.269
PEFR (L/s)†	6.34	5.63, 7.67	-	-	6.79	6.33, 11.57	-	-	6.12	5.63, 7.42	-	-	6.37	5.73, 7.81	-	-	5.52	4.91, 6.12	-	-	0.458
PEFR%†	103.45	88.76, 113.41	-	-	99.27	97.53, 119.28	-	-	100.00	88.06, 108.32	-	-	104.18	93.99, 113.41	-	-	85.09	78.43, 91.75	-	-	0.474
PEFR% < 80%‡	-	-	4	11.4	-	-	0	0	-	-	1	7.7	-	-	2	11.8	-	-	1	50.0	0.358
FEF25-75†	95.42	71.04, 103.50	-	-	64.74	51.59, 101.46	-	-	95.42	83.09, 98.19	-	-	97.05	84.59, 106.67	-	-	66.40	61.75, 71.04	-	-	0.286
FEF25-75‡ < 65%	-	-	7	20.0	-	-	2	66.7	-	-	1	7.7	-	-	3	17.6	-	-	1	50.0	0.095

Note: Data are median (IQR), unless otherwise specified.

FEF25-75, forced expiratory flow at 25% to 75% of the vital capacity; FEV1, forced expiratory volume in one second; FVC, forced vital capacity; PEFR, peak expiratory flow rate; IQR, interquartile range.

* , *p* < 0.05 denotes statistical significance.

† , Continuous variables: Kruskal–Wallis test was used to compare medians across all four cohorts;

‡ , Categorical variables: Chi-squared and Fisher’s exact test were used to compare groups.

In cohort 2, nine of the symptomatic patients had normal FVLs. Two additional ones had a mild restrictive pattern, in one of whom there was also a reduced FEF25% – 75% with a 6% reduction following bronchodilator (40% to 38% predicted). A further two were normal except for a reduced FEF25% – 75% with 29% reversibility following bronchodilator (76% to 97% predicted) in one.

In cohort 3, 13 of the symptomatic patients had normal FVLs, one had an obstructive pattern with a reduced FEF25% – 75% and a 6% change following bronchodilator (58% to 62% predicted), another had an obstructive pattern with no reversibility of FEV1 but a reduced FEF25% – 75% with 29% reversibility following bronchodilator (34% to 45% predicted) and two had restrictive changes.

In cohort 4, one of the symptomatic patients had a normal FVL, and in the other, the FVL was normal, other than having a reduced FEF25% – 75% with 13% reversibility following bronchodilator (63% to 71% predicted).

**FIGURE 2:**
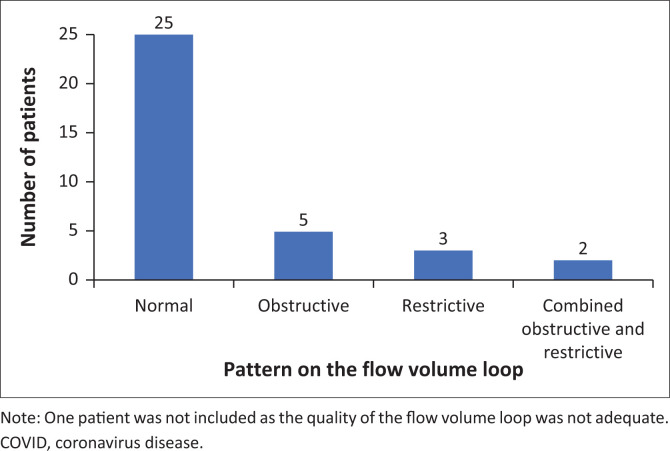
The pattern of the flow volume loop in 35 patients with symptoms of long COVID.

There were no significant differences in the spirometry, either pre- or post-bronchodilator, when comparing the values among the four groups. The pattern of the FVL was normal in 25 patients, obstructive in 5, restrictive in 3 and two had a mixed pattern (Figure 2).

#### Six-minute walk test

The median 6MWD was 445.3 m (IQR: 370.6, 530.0) for the group as a whole and 507.0 (IQR: 415.1, 508.5), 423.9 (IQR: 354.2, 539.5), 455.1 (IQR: 410, 477.0) and 516.0 (IQR: 376.2, 655.8) for cohorts 1, 2, 3 and 4, respectively. The 6MWD was normal in only three patients; nine patients achieved ≥ 80% of predicted, while the remainder had values between 44.7% and 78.8% of predicted.

In most patients, the oxygen saturation remained in a very narrow range during the 6MWT, being either unchanged or increasing or decreasing by ≤ 5%, with all the values remaining above 95% saturation, except in three patients. One patient in cohort 2 and two in cohort 3 had significant decreases in saturation. None of these cases had CT scan changes potentially compatible with previous COVID-19 infection. The 6MWD of cases with CT scan changes potentially associated with COVID-19 lung disease were 212.9 m, 814.0 m, 481.2 m, 410.0 m, 462.0 m, 286.0 m and 354.7 m, respectively (Table 3).

**TABLE 3:** Clinical and lung function parameters in patients with long COVID symptoms and compatible changes on HRCT.

Sex	Age (years)	BMI (kg/m^2^)	Major symptoms	mMRC	6MWT (m)	Spirometry results	HRCT scan of chest	Classification
F	62	56.08	Short of breath	1	212.9	Restrictive with abnormal FEF25%-75%	Mild diffuse fibrosis	Possible
F	75	26.64	Depression and anxiety	1	410.0	Normal	Fibrosis right base possibly post-infective	Possible
F	60	40.73	Short of breath, temperature, cough, sore throat	1	462.0	Abnormal FEF25%-75%	Mild lower lobe reticulation -?early fibrosis, mild bilateral bronchiolectasis especially LL, mild bilateral GG nodules in UZ	Possible
F	48	44.73	Tired/fatigue, short of breath, muscle aches	3	286.0	Restrictive	Diffuse GG attenuation bilaterally, dilatation of bronchi in LL post., bilateral small subpleural band at left base, bulla in RUL	Probable
M	53	27.16	Muscle aches, headache, “brain fog”	1	354.7	Normal	Linear bands of fibrosis in ant. segment of UL, fibrosis in LL, bilateral minor GG opacities LZ	Probable
F	64	33.39	Short of breath	1	814.0	Normal	Few foci of nodular pleural thickening in RLL, possible sparse GG nodules on left	Possible
F	60	30.03	Headaches	1	481.2	Normal	GG attenuation and fibrotic bands peripherally and bilaterally, small nodule in apex	Probable

6MWT, 6-minute walking test distance in meters; ?, possible; BMI, body mass index; CT, computerised tomographic; F, female; GG, ground glass; HRCT, high resolution computerised tomographic; LL, lower lobe; LZ, lower zone; M, male; mMRC, modified Medical Research Council Dyspnoea Scale; RLL, right lower lobe; UZ, upper zone; RUL, right upper lobe; UL, upper lobe; FEF, forced expiratory flow.

#### High-resolution computerised tomographic scanning

Eleven chest HRCT scans were initially reported as being abnormal. Two had abnormalities that were not related to long COVID. A further HRCT was re-evaluated and confirmed to be normal. Among the remaining eight cases, the HRCT changes were thought to be unrelated to long COVID in one, while four had possible and three probable long COVID changes. All the participants in whom the HRCT changes were compatible with long COVID were in cohort 3 except for one in cohort 2 whose HRCT showed changes possibly because of long COVID. Positive HRCT scan findings were significantly more common in cohort 3 (*p* = 0.045). Spirometry was normal in 2 with probable and 1 with possible long COVID changes and was abnormal in 3 with possible and 1 with probable long COVID changes. The 6MWT was abnormal in all but one patient with possible long COVID changes (Table 3).

## Discussion

In this sub-study of pulmonary manifestations in patients with long COVID symptoms, a median of 783 days (IQR: 639–962 days)/median of 2.1 years (range 1–7 to 2.7 years) following a PCR-confirmed or rapid antigen test-positive COVID-19 infection, the main positive findings were as follows: the rate of long COVID was 21.1% overall, with the least number of cases in those with previous asymptomatic infection (5.9%) or who had prior vaccination (10.5%), while the highest rate was in those with previous severe infection (37.8%)(*p* = 0.001); the overall BMI was high (mean ± standard deviation, 36.2 ± 9.3 kg/m^2^) with a non-significant trend for this to be highest in cohort 3; the most common comorbidities were hypertension and diabetes mellitus with a non-significant trend for hypertension to be more common in cohort 3; the mMRC score was ≥ 1 in 24 patients, most frequently in cohort 3 (*p* = 0.30); and a total of eight HRCT scans were abnormal, most commonly in cohort 3 (7 cases; *p* = 0.045) of which four were considered to be possibly and three probably related to COVID-19.

Previous studies have reported the incidence of long COVID to be between 10% and 30% in previously non-hospitalised patients, more frequent in those previously hospitalised and less frequent (10% – 12%) in those previously vaccinated.^26^ A conservative estimate has suggested that ~10% of patients with acute COVID-19 will develop long COVID, and globally, it has been estimated that there are approximately 65 million people living with this condition although this is likely considerably underestimated.^26^ The differences in the incidences reported in the vast literature on the topic are because of different definitions used, the presence of multiple putative pathophysiological mechanisms, a lack of any solitary, reliable biomarker used for diagnosis, monitoring or research, the subsequent evolution of the natural history of the infection because of changes in the virus and the introduction of novel therapies and vaccination.^1^

The finding in this study of an overall incidence of self-reported long COVID of 21.1% is consistent with the literature.^1^ Similar to that previously described, the lowest number of cases were found in the cohorts with previous asymptomatic infection or prior COVID vaccination and highest among those with more severe previous infection (*p* = 0.001).

The prevalence of long COVID reported in any study depends on the definition used; there is relatively poor consensus on which definition is most appropriate, and definitions have evolved over time.^1,27,28^ The definition used in this study was that of the WHO,^18^ which was one of the earliest and was the most appropriate choice at the time this study was started.

The median age of our patients was 47 years, compatible with that reported previously, with publications indicating that the highest percentage of cases occur between 35 and 50 years, and others indicating that while there is an inverted u-shaped age incidence, most cases occur in middle age.^1,28^ There were four males and 32 females, which is also compatible with publications that have shown that females experience a higher prevalence of self-reported long COVID than males, a finding that is independent of demographic and clinical characteristics.^1,28,29^ Importantly, at least some of the vast differences noticed among the various parameters in different studies represents the widely divergent population sampling frames.^1^

Characteristics such as high BMI, as well as underlying comorbidities, increase the risk of long COVID.^1,28,29^ The mean BMI was very high in this study, and while it was highest in the group that had previously had severe COVID-19, this did not reach significance. High BMI is not only a risk factor for severe COVID-19 infection^30,31,32^ (a BMI of more than 23 kg/m^2^ is associated with a linear increase in risk)^33^ but also for long COVID.^34^

Rather uniquely in this study, we excluded cases with pre-existing respiratory comorbidities or respiratory risk factors, to avoid possible confounding with any respiratory abnormalities that would be documented. While this resulted in the recruitment of a smaller number of patients than we anticipated, there was nevertheless still a relatively high incidence of long COVID in the included participants.^1^

Symptoms of long COVID are heterogenous and multisystemic and can change over time.^1^ The most common symptoms in our patients were tiredness and/or fatigue, shortness of breath and cough, and these are among the most common respiratory symptoms noticed in long COVID studies.^1^

Various abnormalities occur in lung function parameters in patients post-COVID-19.^9,35,36,37,38,39,40^ An early review suggested that while spirometric indices were usually well preserved, defects in DLCO occurred commonly, in up to 20% – 30%, following mild to moderate disease, and in 60% following severe infection.^35^ Unfortunately, in the current study, we were not able to do full lung function studies with measurement of DLCO.

A meta-analysis of seven studies of patients with previous COVID-19 infection documented that within 3 months following hospital discharge, 22.9% had abnormal spirometry^9^; 15% showed restriction, while 7.9% showed obstruction. Another study at 2 months post-discharge reported spirometric abnormalities in 17.2% following mild-to-severe COVID-19, of which an obstructive pattern was slightly more common than a restrictive pattern.^39^ In a study of 46 patients followed for up to 12 months post-hospital discharge, restrictive and obstructive defects were documented in 13 and three patients, respectively, with 10 having a combined defect.^41^ Spirometric abnormalities in the current cohort included obstructive changes most commonly, followed by an almost equal numbers of cases with restrictive and combined changes.

In several of the current patients, abnormalities in FEF 25% – 75% were documented, as has been observed in other studies,^42^ even in the presence of normal FEV1, FVC and FEV1/FVC ratio. Studies have suggested that these findings may represent patients with small airways disease, or even undiagnosed asthma or early COPD,^43,44,45^ although there is still some debate about the true value of such measurements. We only reported the FEF 25% – 75% values as abnormal if the absolute values were < 65% predicted and/or if the percentage predicted changes following bronchodilator were ≥ 0.87 L/s and 27%, as suggested in several publications previously.^42,43,46^ Nevertheless, as we had specifically excluded patients with other potential causes for these findings on spirometry, we initially questioned whether these abnormalities may have been a specific manifestation of long COVID. However, in contradiction to this consideration was the fact that several asymptomatic control patients also manifested these similar abnormalities in FEF25% – 75% (data not shown), making this possibility highly unlikely. There were no apparent relationships of the various lung function tests with each other, and although more abnormalities were noticed in cohort 3, this also did not reach significance.

The 6MWT has been affirmed in an official ERS/ATS systematic review to be a valid and reliable measure of exercise capacity for people with chronic lung disease, being a test more of functional exercise performance rather than lung function.^25,47^ Notably, physical factors, such as morbid obesity, may impact on its performance, which needs consideration in this study. Overall, the 6MWD was reduced in all but three patients and ≥ 80% of predicted in only nine. Persistent reductions have previously been found to be closely related to previous severe COVID-19 infection or the occurrence of COVID-19 pneumonia.^37^ Furthermore, while the occurrence of hypoxaemia during the 6MWT has been noted previously to be a potential marker of a reduced gas transfer this only occurred in three cases (using a cut-off value of 5%) in this study.^48^ One aspect of the performance of the 6MWT in this study that needs consideration, is that because of a restriction of available space, rather than having a single track of 30 m, with only one turn per circuit for each patient, four tracks of 15 m were used. Availability of space has previously been noticed to be the most common reason for adapting the 6MWT in low resource settings and may lead to an underestimation of the patients’ abilities.^25,49,50^

Studies have documented numerous changes on the chest HRCT scan in patients with long COVID, the most frequently used additional chest imaging modality.^51,52,53^ However false-negative scans do occur, and false-positive scans may be because of other respiratory conditions causing changes that mimic long COVID.^52,53^ The different imaging findings have been characterised into interstitial, pleural, airway abnormalities and other parenchymal findings or as typical, intermediate, atypical and negative findings.^51,52,53^ Seven of our patients had abnormalities potentially related to long COVID. Importantly, changes in HRCT scan following mild-to-moderate COVID-19 do not necessarily correlate with symptoms, as seems to be the case in our patients.^54^

While this is among the first studies documenting in some detail the pulmonary characteristics of patients with long COVID in South Africa, there are potential limitations. Firstly, this was a single centre study, so that the findings may not be generalisable to other institutions or geographical areas. Secondly, while the initial study had a relatively large number of cases (352 patients), only 36 symptomatic patients were enrolled into this study, which may be ascribed to several reasons, including the fact that several cases were excluded to avoid confounding from existing pulmonary conditions or risk factors. The low number of cases with long COVID was of particular concern for any analysis including cohort 4 (previously vaccinated cases), as there were only two cases, which were also very different (e.g. one had a normal BMI and the other a BMI of 39 kg/m^2^). This may have limited the finding of significant differences in the data among the four cohorts although there was, nevertheless, still a trend for findings to be more frequent in those with previous severe COVID-19 infection. With regard to lung function testing, including both spirometry (especially, FVC and FEF25% – 75%) and the 6MWT, it is important to recognise that the high BMIs found in many of the patients may have impacted the reported findings. Furthermore, although additional lung function testing had been planned, these were not performed because of cost. Thirdly, because of space constraints, the method of measuring the 6MWT was modified, as described above, and may have underestimated the actual distance the participants were able to walk.

## Conclusion

This study indicated that there were pulmonary abnormalities that occurred in some patients as long as 2 to 3 years after confirmed SARS-CoV-2 infection. The fact that often there are no measurable or documentable abnormalities specifically associated with long COVID symptoms suggests that an objective diagnostic test, such as a universal biomarker, needs to be sought to validate the true burden of symptoms suffered by those affected. Confirmed long COVID needs comprehensive evaluation, as well as further assessment and management, as recommended in Respiratory Society Guidelines.^55^
